# Can contrast-enhanced ultrasound differentiate the type of hepatic echinococcosis: cystic echinococcosis or alveolar echinococcosis?

**DOI:** 10.1186/s13071-023-05731-2

**Published:** 2023-04-17

**Authors:** Xuhui Zhang, Lamu Suolang, Yelei Ren, Yifei Wang, Yong Jiang, Xiaofei Zhong, Zehui Gou, Wu Zhou, Juan Chen, Yongzhong Li, Diming Cai

**Affiliations:** 1 Department of Medical Ultrasound, West China Hospital, Sichuan University, Chengdu, 610041 China; 2 Center of Disease Control and Prevention, Tibet Autonomous Region, Lhasa, 850002 China; 3 Department of Pathology, West China Hospital, Sichuan University, Chengdu, 610041 China

**Keywords:** Conventional ultrasound, Contrast-enhanced ultrasound, Hepatic echinococcosis, Cystic echinococcosis, Alveolar echinococcosis, Differentiation

## Abstract

**Background:**

Hepatic echinococcosis (HE) is a zoonotic disease caused by *Echinococcus*, and *Echinococcus granulosus* and *E. multilocularis* are the most common, causing cystic echinococcosis (CE) and alveolar echinococcosis (AE), respectively. Contrast-enhanced ultrasound (CEUS) is an imaging technique which has been recommended for identifying focal lesions in the liver. However, the effect of CEUS on the differentiation of hepatic echinococcosis type remains unclear.

**Methods:**

Twenty-five patients with 46 HE lesions confirmed by histopathology in our hospital from December 2019 to May 2022 were reviewed by conventional ultrasound (US) and CEUS examinations, respectively. After US was completed, the CEUS study was performed. A bolus injection of 1.0–1.2 ml of a sulfur hexafluoride-filled microbubble contrast agent (SonoVue^®^) was administered. The images and clips of the lesions by US and CEUS were reviewed retrospectively. The lesions detected using US were evaluated including the location, size, morphology, margin, internal echogenicity and the internal Doppler signal. The lesions detected using CEUS were evaluated including the enhancement degree, enhancement pattern and enhancing boundary in different phases. The diagnoses of lesions by US or CEUS were respectively recorded. By taking the histopathology as the gold standard, the paired Chi-square test was performed with statistical software (IBM SPSS; IBM Corp., Armonk, NY, USA), and the results of differentiation of HE type by US and CEUS were statistically analyzed.

**Results:**

A total of 46 lesions were involved in 25 patients, including 10 males (40.0%) and 15 females (60.0%) aged 15–55 (42.9 ± 10.3) years. By histopathology, 24 lesions of nine patients were diagnosed as CE and 22 lesions of 16 patients were diagnosed as AE. Among the 46 HE lesions, compared with histopathological examination, the accuracy rate was 65.2% and 91.3% in US and CEUS findings, respectively. Among the 24 CE lesions, 13 lesions were correctly differentiated by US, and 23 by CEUS. The difference between US and CEUS was statistically significant (Chi-square test, ${\chi }^{2}$ = 8.10, *df* = 23, *P* < 0.005). Among the total 46 HE lesions, 30 lesions were correctly differentiated by US, and 42 by CEUS. The difference between US and CEUS was statistically significant (Chi-square test, ${\chi }^{2}$ = 10.08, *df* = 45, *P* < 0.005).

**Conclusions:**

CEUS is a more effective technique than US for differentiating the type of HE between CE and AE. It could be a reliable tool in the differentiation of HE.

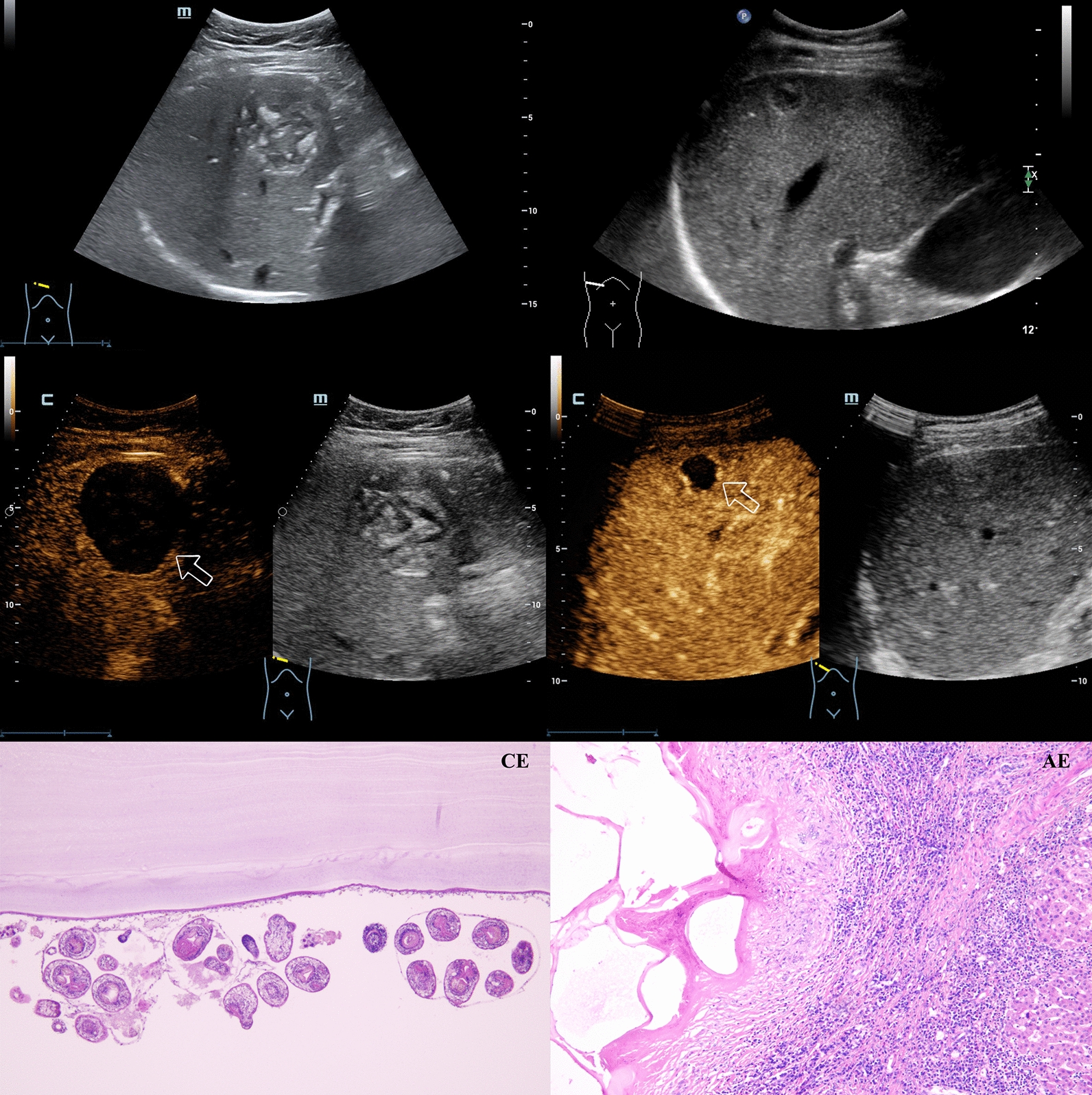

## Background

Hepatic echinococcosis (HE) is an infection of the liver caused by species of *Echinococcus*, of which *Echinococcus granulosus* and *E. multilocularis* are the most common, causing cystic echinococcosis (CE) and alveolar echinococcosis (AE), respectively. The World Health Organization (WHO) recommends percutaneous treatment, drug therapy, surgery or “watch and wait” for CE [1–3]. Surgery is the preferred choice of treatment for AE, with radical resection of the entire parasitic lesions in the liver being the optimal treatment [4]. The prognosis of AE is worse than CE. It is reported that without treatment or with improper treatment, the mortality rate for CE is about 2–4%, whereas more than 90% of patients with AE will die within 10 years after the onset of clinical symptoms, and almost 100% of patients will die within 15 years [5–7]. Thus, different types of HE are associated with different prognoses, and therefore accurate differentiation of HE type is important for patients with HE. Conventional ultrasound (US) is recommended by WHO as the preferred medical imaging to diagnose HE. However, it is difficult to differentiate some HE lesions in US, especially between CE4 or CE5 and AE [8]. Contrast-enhanced ultrasound (CEUS) is an imaging tool used for the diagnosis of hepatic nodules. CEUS has overcome the limitations of US and can visualize the parenchymal microvasculature. It can thus provide more information than US for the diagnosis of hepatic nodules [9]. In the present study, we evaluated the value of CEUS in differentiating the type of HE.

## Methods

### Patient data

The results of US and CEUS examinations in 25 patients with 46 HE lesions, who were admitted to our hospital between December 2019 and May 2022, were reviewed retrospectively. This study was approved by the ethics committee of our hospital. All patients underwent surgery, and the diagnoses were confirmed histopathologically.

### Ultrasound examination

The US and CEUS examinations were performed using Philips iU22 (convex probe, C5-1 MHz; Mountain View, CA, USA) and Mindray Resona 7 (convex probe, SC6-1 MHz; Shenzhen, Guangdong, China) US scanners. The US systems were equipped with a harmonic contrast pulse sequencing apparatus. The contrast agent used was SonoVue^®^ (Bracco Medical Imaging Deutschland GmbH, Konstanz, Germany) and the suspension contained stabilized sulfur hexafluoride microbubbles. All patients were asked to fast for 8 h before US examination. After US was completed, the CEUS study was performed. CEUS was started at a low mechanical index of 0.06. SonoVue^®^ suspension (1.0–1.2 ml) was administered intravenously via an indwelling vein cannula within 2 s, followed by a flush with 5.0 ml saline solution. Patients who failed to obtain satisfactory images received 1.0–1.2 ml SonoVue^®^ again for better visualization of the reference lesion after 10-min intervals. As previously described by Schweizer et al. [10], the period of 7–30 s post-injection (p.i.) was defined as the arterial phase, 31–120 s p.i. as the portal phase and 121–360 s p.i. as the late phase. The target lesion and surrounding liver parenchyma were observed continuously for 5 min. The entire CEUS examination was stored as a dynamic digital video file on the hard disk of the US system and recorded on a digital video recorder.

### Image evaluation

The ultrasonic imaging data of the patients were extracted from the databases, and the diagnoses were made based on the features of the lesions in US and CEUS images. The location, size, morphology, margin and internal echogenicity of the lesions were observed and recorded by US. According to the WHO classification criteria for CE in US [11] and expert consensus on the diagnosis and treatment of HE [7], lesions with a circumscribed margin, regular shape and homogeneous echogenicity, as well as the presence of the double cystic wall or snowflake sign (CE1), rosette-like or honeycomb-like sign (CE2), water-lily sign (CE3), ball of wool sign (CE4) or eggshell calcified wall sign (CE5), were diagnosed as CE. According to the WHO classification criteria for AE in US [1] and the classification criteria proposed by Kratzer et al. [12], lesions with an ill-defined margin, irregular shape, heterogeneous echogenicity and scattered calcification foci, as well as the presence of hailstorm sign, pseudocystic sign, hemangioma-like sign, ossification sign or metastasis-like sign, were diagnosed as AE.

CEUS images were reviewed to observe and record the enhancement degree, enhancement pattern and enhancing boundary of the lesions in different phases. By comparison with the surrounding normal hepatic parenchyma, the enhancement degree of the lesions was classified as non-enhancement, hypo-enhancement, iso-enhancement and hyper-enhancement. The enhancement patterns were classified as homogeneous, heterogeneous and rim enhancement. The enhancing boundary was classified as circumscribed boundary and ill-defined boundary. The lesions with non-enhancement and enhancing circumscribed boundary were diagnosed as CE, while the lesions with hyper-enhancement, heterogeneous or rim enhancement and enhancing ill-defined boundary were diagnosed as AE.

### Statistical analysis

Taking the results of histopathology as the gold standard, the paired Chi-square test was performed with SPSS version 26.0 software (IBM Corp., Armonk, NY, USA), and the results of US and CEUS were statistically analyzed. A value of *P* < 0.05 was considered statistically significant.

## Results

### General information

A total of 25 patients were investigated, including 10 males (10/25, 40.0%) and 15 females (15/25, 60.0%) aged 15 to 55 (42.88 ± 10.28) years. There were 46 lesions with sizes (diameter) of 1.10 to 15.00 (7.12 ± 2.79) cm. Seventeen single lesions were found in 17 patients and 29 multiple lesions were found in eight patients. The lesions were located in the right lobe in 18 patients with 28 lesions, in the left lobe in eight patients with 12 lesions, in the porta hepatis in two patients with two lesions, at the junction of the left and right lobes in three patients with three lesions, and in the post-mediastinum in one patient with one lesion (Table 1).

**Table 1:** Overview of patients with HE lesions

Total (*N* = 25)	Frequency (%)
Age (years)	
Mean ± SD (median)	42.88 ± 10.28 (43.00)
Min–max	15.00–55.00
Gender	
Male	10 (40.00%)
Female	15 (60.00%)
Number of lesions (NoL)	
1	17 (68.00%)
2	2 (8.00%)
3	4 (16.00%)
4	1 (4.00%)
9	1 (4.00%)
Lesion size (mm)	
Mean ± SD (median)	7.12 ± 2.79 (5.95)
Min–max	1.10–15.00
Localization of lesions	
Right lobe (NoL)	18 (28, 60.87%)
Left lobe (NoL)	8 (12, 26.09%)
Porta hepatis (NoL)	2 (2, 4.35%)
Junction of left and right lobes (NoL)	3 (3, 6.52%)
Post-mediastinum (NoL)	1 (1, 2.17%)
Number of CE lesions	
CE1 (NoL)	3 (5, 20.83%)
CE2 (NoL)	2 (3, 12.50%)
CE3a (NoL)	1 (1, 4.17%)
CE3b (NoL)	1 (1, 4.16%)
CE4 (NoL)	3 (10, 41.67%)
CE5 (NoL)	4 (4, 16.67%)
Total (NoL)	9 (24, 100.00%)
Number of AE lesions	
Hailstorm sign (NoL)	4 (5, 22.72%)
Pseudocystic sign (NoL)	6 (6, 27.27%)
Hemangioma-like sign (NoL)	7 (9, 40.91%)
Ossification sign (NoL)	1 (1, 4.55%)
Metastasis-like sign (NoL)	1 (1, 4.55%)
Total (NoL)	16 (22, 100.00%)

*HE* hepatic echinococcosis, *SD* standard deviation, *CE* cystic echinococcosis, *AE* alveolar echinococcosis

### US findings and diagnosis

Among the 24 CE lesions, 13 lesions were correctly diagnosed, of which five were CE1, three were CE2, two were CE3, two were CE4 and one was CE5, and 11 lesions were diagnosed incorrectly, of which eight were CE4 and three CE5.

Among the 22 AE lesions, 17 lesions were correctly diagnosed, of which five showed a hailstorm sign, five showed a pseudocystic sign, five showed a hemangioma-like sign, one showed an ossification sign and one showed a metastasis-like sign, and five lesions were diagnosed incorrectly, of which one showed a pseudocystic sign and four showed a hemangioma-like sign.

**Fig. 1:**
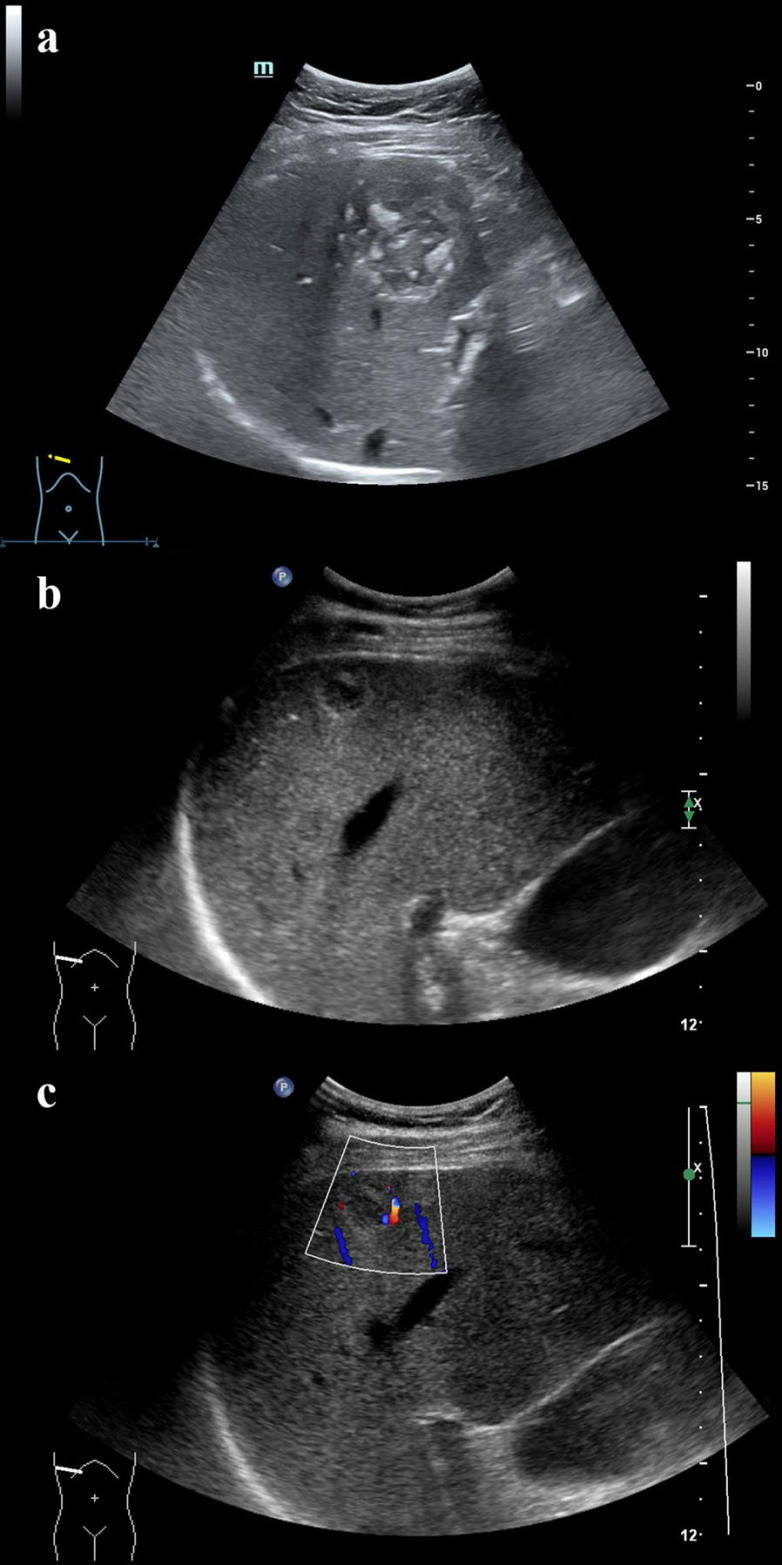
The US ultrasonogram of CE and AE. **a** A CE lesion exhibited circumscribed margin, regular shape and heterogeneous echogenicity with scattered calcification foci. **b** An AE lesion exhibited ill-defined margin, regular shape and heterogeneous echogenicity. **c** color Doppler ultrasonogram corresponding to **b** showed tiny punctiform and linear blood flow signals inside the lesion. *US* conventional ultrasound, *CE* cystic echinococcosis, *AE* alveolar echinococcosis

Among the 46 lesions of HE, 30 lesions were correctly diagnosed and 16 lesions were incorrectly diagnosed in US, with an accuracy rate of 65.22% (Table 2, Fig. 1).

**Table 2:** Comparison of the findings of US with histopathological examination

US	Histopathology		Total
	CE (*n*)	AE (*n*)	
CE (*n*)	13	5	18
AE (*n*)	11	17	28
Total	24	22	46

*US* conventional ultrasound, *n* number, *CE* cystic echinococcosis, *AE* alveolar echinococcosis

### CEUS findings and diagnosis

**Fig. 2:**
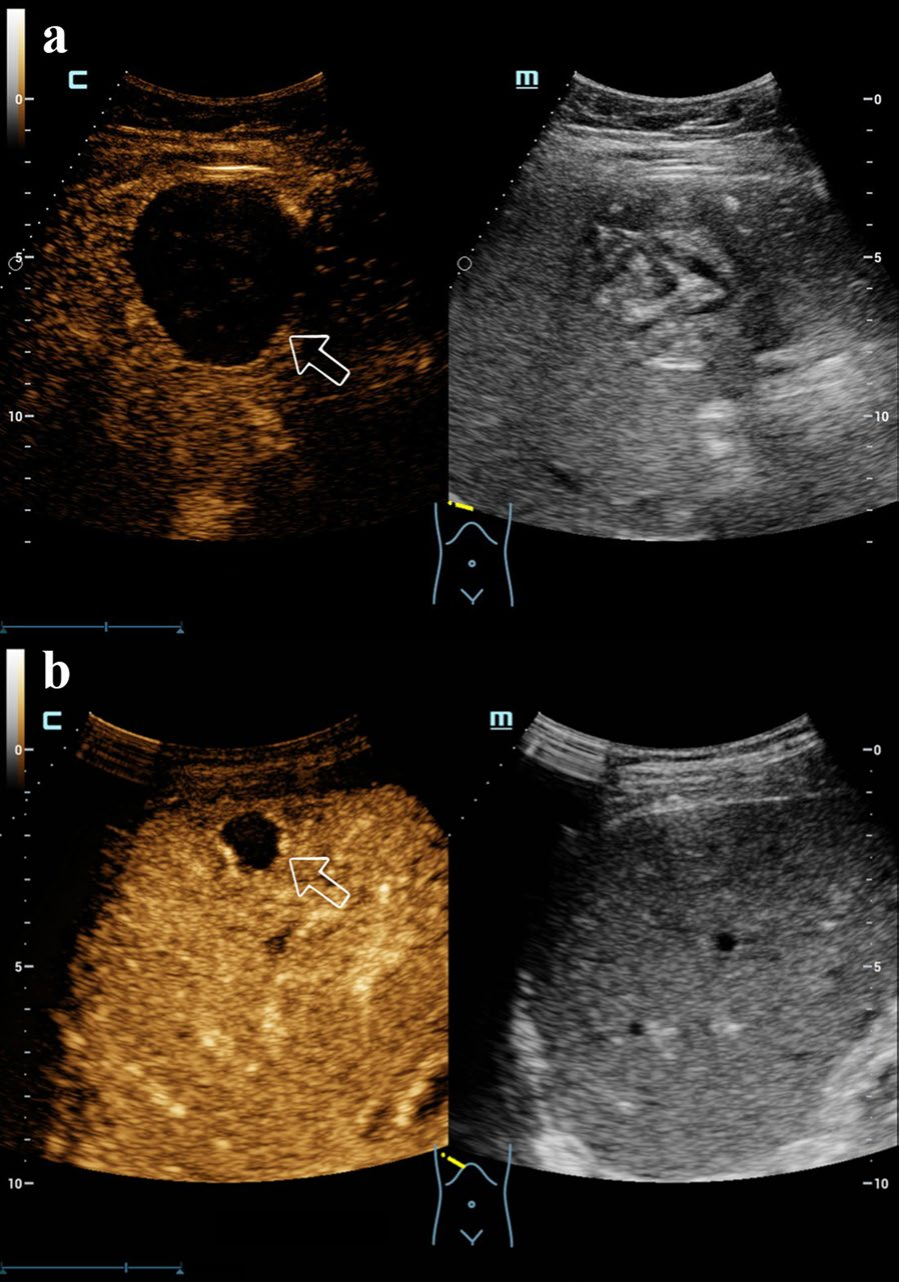
The CEUS ultrasonogram of CE and AE. **a** A CE lesion showed enhancing circumscribed boundary and non-enhancement in the arterial phase (white arrow), indicating that the lesion may not invade the parenchyma of the surrounding normal liver. **b** An AE lesion showed enhancing ill-defined boundary and hyper-enhancement in the arterial phase, whose enhancement pattern was the circular ring of enhancement with non-enhancement areas within the lesion (white arrow), suggesting that there may be inflammatory reaction zones around the lesion and the lesion may contain marginal microvessels with more abundant blood supply. *CEUS* contrast-enhanced ultrasound, *CE* cystic echinococcosis, *AE* alveolar echinococcosis

Among the 46 lesions of HE, 42 lesions were correctly diagnosed and four lesions were incorrectly diagnosed in CEUS, with an accuracy rate of 91.30% (Table 3, Fig. 2).

**Table 3:** Comparison of the findings of CEUS with histopathological examination

CEUS	Histopathology		Total
	CE (*n*)	AE (*n*)	
CE (*n*)	23	3	26
AE (*n*)	1	19	20
Total	24	22	46

*CEUS* contrast-enhanced ultrasound, *n* number, *CE* cystic echinococcosis, *AE* alveolar echinococcosis

### Comparison of the results of US with CEUS in the diagnosis of CE

Among the 24 lesions of CE, 13 lesions were correctly diagnosed and 11 lesions were misdiagnosed by US, while 23 lesions were correctly diagnosed and one lesion was misdiagnosed by CEUS (Chi-square test, ${\chi }^{2}$ = 8.10, *df* = 23, *P* < 0.005). (Table 4).

**Table 4:** Comparison of the results of US with CEUS in the differentiation of CE

US	CEUS		Total
	Positive (*n*)	Negative (*n*)	
Positive (*n*)	13	0	13
Negative (*n*)	10	1	11
Total	23	1	24

*US* conventional ultrasound, *CEUS* contrast-enhanced ultrasound, *n* number, *CE* cystic echinococcosis

### Comparison of the results of US with CEUS

Among the 46 lesions of HE, 30 lesions were correctly diagnosed and 16 lesions were incorrectly diagnosed by US, while 42 lesions were correctly diagnosed and four were misdiagnosed by CEUS (Chi-square test, ${\chi }^{2}$ = 10.08, *df* = 45, *P* < 0.005) (Table 5).

**Table 5:** Comparison of the results of US with CEUS in the differentiation of HE

US	CEUS		Total
	Positive (*n*)	Negative (*n*)	
Positive (*n*)	30	0	30
Negative (*n*)	12	4	16
Total	42	4	46

*US* conventional ultrasound, *CEUS* contrast-enhanced ultrasound, *n* number, *HE* hepatic echinococcosis

### Histopathological findings

**Fig. 3:**
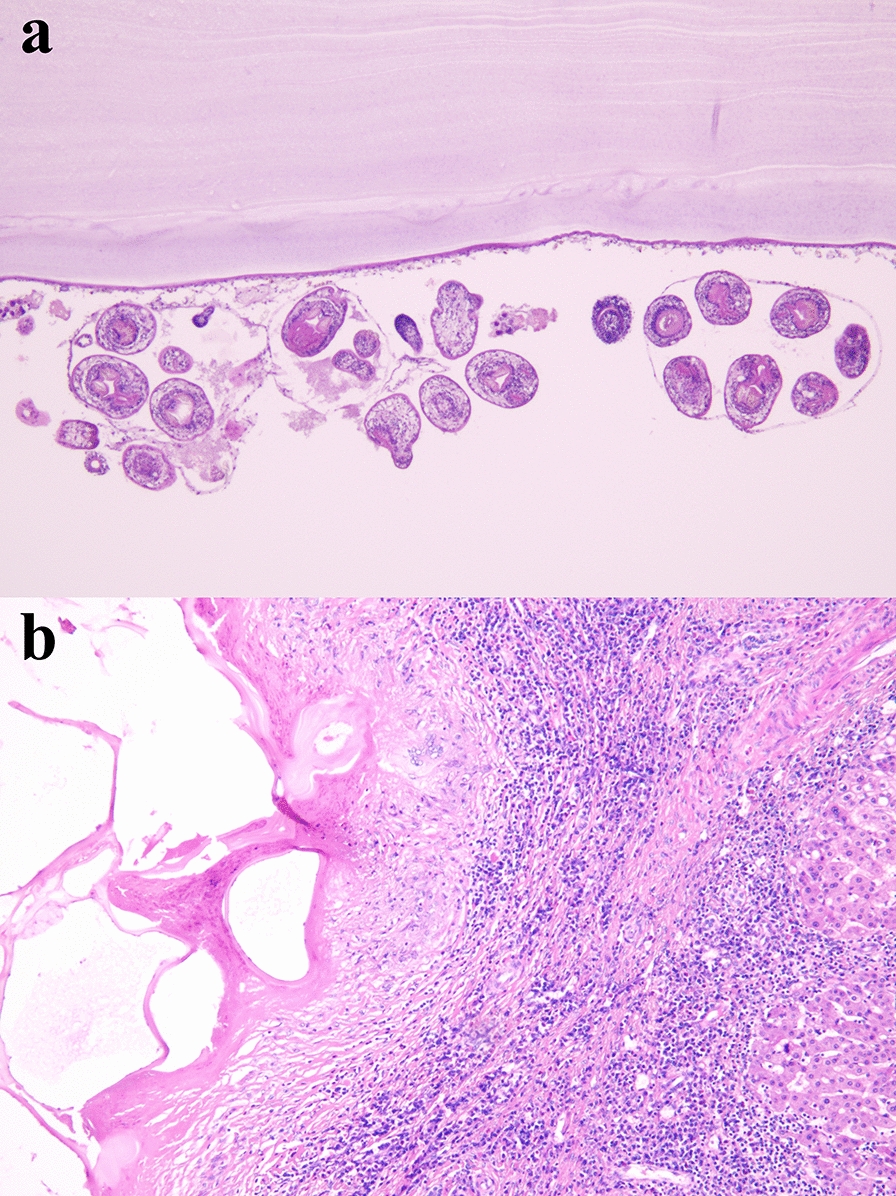
H&E staining results for CE and AE. **a** Powdery cortex, germinal layer and hexacanth were visible for CE; **b** Powdery cortex, necrosis and granulomas were visible for AE (magnification, both ×100). *H&E* hematoxylin and eosin, *CE* cystic echinococcosis, *AE* alveolar echinococcosis

As confirmed by histopathology, 24 lesions of nine patients were diagnosed as CE and 22 lesions of 16 patients were diagnosed as AE (Fig. 3).

## Discussion

Echinococcosis is a neglected zoonotic disease caused by a parasite of the genus *Echinococcus* within Cestoda, and is associated with damage or dysfunction of target organs, particularly the liver (70%). Western China is one of the most endemic regions for CE and AE, with the main risk factors being exposure to dogs and the raising of livestock [6, 13].

AE and CE are two completely distinct diseases [14], with significant differences in the morphology, epidemiology, histopathology, clinical course, clinical management and prognosis. CE shows expansive growth, and percutaneous treatment or albendazole is recommended for treating the disease [3, 7]. Known as “worm carcinoma,” AE shows an infiltrative growth with features similar to malignancy and can develop distant metastasis [15–17]. Obviously, AE is more harmful to health and requires surgery or close follow-up. Therefore, the correct differentiation of CE and AE is particularly important in the prognosis and treatment of patients.

CE1, CE2, CE3a and CE3b feature unique ultrasonic characteristics, including the double cystic wall or snowflake sign for CE1, rosette-like or honeycomb-like sign for CE2, water-lily sign for CE3a, complex mass sign for CE3b and so on, easy to diagnose by US. However, the sonographic features of CE4 and CE5 in US are difficult to differentiate from AE [18]. At these stages, CE4 appears as a coarse variable (hyper or hypo)-echogenic echotexture without daughter vesicles, showing the ball of wool sign. CE5 appears as partially or completely calcified with shadowing, showing the eggshell calcified wall sign. AE lesions, especially the hemangioma-like subtype, present hyperechoic features in comparison with the surrounding hepatic parenchyma. Therefore, the definitive diagnosis between CE4 or CE5 and AE usually cannot be made by US findings alone, and our study attempted to differentiate between the two using CEUS [19]. In this study, 16 lesions were incorrectly diagnosed in US, of which eight lesions were CE4, three were CE5, one showed a pseudocystic sign and four showed a hemangioma-like sign. A retrospective analysis of the misdiagnosed lesions revealed that the ultrasonographic features in US of some CE4 or CE5 lesions were highly similar to those of AE lesions with a hemangioma-like sign, both of which exhibited slightly hyperechoic or strongly hyperechoic features with coarse margins.

CEUS is a novel imaging modality that has been recommended for the identification of focal hepatic lesions. It overcomes the limitations of the conventional grayscale US and color or energy Doppler US, and allows real-time visualization of the microvasculature within the parenchyma [20–22]. Studies have shown that CEUS is more conducive to the diagnosis and differentiation of AE than US [23, 24]. Most AE lesions show hyper-enhancement in the arterial phase, with gradual fading in the portal and delayed phases [25]. The most common enhancement pattern of AE lesions is the circular ring of enhancement [25], with areas of non-enhancement within the lesion [23, 26]. Some studies have referred to this phenomenon as the “black hole sign” [10, 27]. Due to the budding or infiltrative proliferation of AE, new vesicles are constantly produced and penetrate into surrounding tissues, similar to malignancy. AE lesions can not only invade the adjacent tissue structures directly, but also metastasize via lymphatic and hematological routes to the retroperitoneum and distant organs such as the brain and lungs [15]. Therefore, in CEUS imaging, the rim enhancement band may indicate that the AE lesions contain marginal microvessels with more abundant blood supply, laying the foundation for the infiltration and reproduction of *E. multilocularis* [17]. Meanwhile, the enhancing ill-defined boundary may suggest the inflammatory reaction zones around the AE lesions [16]. Due to the expansive growth of CE, lesions mainly cause physiological compression on the surrounding hepatic tissues and main intrahepatic ducts [7]. Therefore, in CEUS imaging, the ultrasonogram of CE lesions mainly shows non-enhancement and enhancing circumscribed boundary without rim enhancement. In this study, four lesions were incorrectly diagnosed in CEUS, of which one lesion was CE4, one showed a hailstorm sign, one showed a pseudocystic sign and one showed an ossification sign. A retrospective analysis of the misdiagnosed lesions revealed that the enhancement features of the CE4 lesion were atypical and crossed with AE, including heterogeneous rim enhancement and enhancing ill-defined boundary, and the other misdiagnosed AE lesions showed non-enhancement or enhancing circumscribed boundary, which were difficult to diagnose.

CT and magnetic resonance imaging (MRI) examination have the advantages of a multi-angle, multi-parametric and high-definition protocol. The location of the focus and its relationship with blood vessels and biliary tracts can be displayed in many directions, which can more accurately assess vascular and biliary complications. It is extremely important for selecting treatment plans, designing operation modes and predicting surgical risk [7, 28, 29]. Since Kodama et al. [30] classified AE into five subtypes by MRI, it is suggested that the characteristics of AE lesions in CEUS may depend on the subtype of AE.

## Conclusions

In conclusion, CEUS is a reliable tool in the differentiation of HE. Compared with US, CEUS is more accurate in differentiating HE and CE. The lesions with non-enhancement and enhancing circumscribed boundary should be suspected as CE, while the lesions with hyper-enhancement, heterogeneous or rim enhancement and enhancing ill-defined boundary should be suspected as AE. However, due to the retrospective nature of this study and the small sample size, as well as the atypical enhancement patterns of some CE and AE lesions in CEUS, further studies are needed to validate the value of CEUS in the differentiation of the type of HE.

## Data Availability

All relevant data are included in the figures and tables of the manuscript.

## Abbreviations

HE: Hepatic echinococcosis
CE: Cystic echinococcosis
AE: Alveolar echinococcosis
US: Ultrasound
CEUS: Contrast-enhanced ultrasound
